# A practical approach for colorectal cancer diagnosis based on machine learning

**DOI:** 10.1371/journal.pone.0321009

**Published:** 2025-04-29

**Authors:** Nguyen Hai Minh, Tran Quang Quy, Ngo Duc Tam, Tran Manh Tuan, Le Hoang Son

**Affiliations:** 1 Thai Nguyen University, Information and Communication Technology, Thai Nguyen, Vietnam; 2 Artificial Intelligence Research Center, VNU Information Technology Institute, Vietnam National University, Hanoi, Vietnam; 3 Faculty of Computer Science and Engineering, Thuyloi University, Hanoi, Vietnam; Newcastle University, UNITED KINGDOM OF GREAT BRITAIN AND NORTHERN IRELAND

## Abstract

In this paper, we present the results of applying machine learning models to build a Colorectal Cancer Diagnosis system. The methodology encompasses six key steps: collecting raw data from Electronic Medical Records (EMRs), revising feature attributes with expert input, data preprocessing, model adaptation, training machine learning models (CART, Random Forest, and XGBOOST), and evaluating the results. Furthermore, based on analysis of experimental measurement parameter values, 21 feature attributes which relate to support early diagnose the Colorectal cancer disease are extracted. Among different models implemented in our case, XGBOOST is the most suitable model to solve this problem. The system assists clinicians to select clinical tests and medical procedures for a colorectal cancer patient. Therefore, patients can save the waiting time and medical examination costs. On the other hand, based on the achievements from this research, our approach can guide further applying machine learning in medicine.

## 1. Introduction

Colorectal colon, with the third highest diagnosis rate, is the second dangerous cancer in Viet Nam. Colorectal cancer constitutes a substantial public health challenge, particularly among men. Originating from malignant cells within the rectum, a segment of the large intestine, colorectal cancer progresses through distinct stages, often asymptomatically during its early phases. Early detection via systematic screening is pivotal for improving clinical outcomes and reducing mortality. However, traditional diagnostic modalities, including endoscopy and pathological biopsy, are hindered by their significant time, cost, and accessibility constraints [1,2]. Diagnosing is one of the core principles of medicine based on the integration of multi-source data analysis and clinician experience. Because of the variety of tumor symptoms, rapid tumor growth, individual differences, and drug sensitivity, it is difficult for doctors to diagnose tumors accurately. Artificial Intelligence (AI) supports clinicians in qualitative diagnosis and detecting the stage of colon cancer, which currently relies on endoscopy and pathological biopsy [3]. Colonoscopy is considered as the gold standard procedure for diagnosing colorectal disease. It is strongly recommended by national societies as an early screening criterion [4–11].

AI technologies in healthcare support doctors in reducing the time and decreasing the overload of work as well. AI tools have been most commonly used in digital technology and data management. AI can manage medical records and other information in different data types. In mobile health, AI helps doctors to diagnose and predict illness. This allows doctors to do the necessary medical interventions before patients need to be hospitalized. It leads to the reduce in costs and fees for both the hospital and the patient. AI also assists the pathologist in accurately reviewing the results using various advanced techniques [12]. In medicine, AI is mainly used to diagnose, treat, and predict disease prognosis. AI mechanisms are divided into two branches, including virtual branch and physical branch. The first includes medical imaging, diagnostic and therapeutic clinical assistant, and drug research. The later includes surgery and nursing [13].

Popular machine learning models, including Artificial Neural Networks (ANN), Multiple Regression (MLR), Bayesian Classification (NBC), Decision Trees (DT), Support Vector Machines (SVM), and K-nearest neighbor (KNN) have been widely used in diagnosis. Decision tree is applied in many healthcare researches [14,15]. In [16], a neural network was applied to create high-quality models to accurately classify all diseases. This research included the experiments on three different types, including diabetes, heart disease and cancer. In addition to that, convolutional neural network (CNN) was used to predict the condition of late-stage cancer or stage 4 cancer and achieve the desired results [17]. Some scientists focused on using ensemble methods to obtain the accurate models and they diagnosed cancer through histopathological examination of the images [18]. In [19,20], multiple machine-learning methods were applied to develop a predictive application for colorectal cancer, including: support vector machines (SVM), k-nearest neighbors (KNN), boosting ensembles, random forests, convolutional neural networks, recurrent neural networks, and recursive neural networks. However, most machine-learning models used for predicting colorectal cancer primarily focused on the later stages of the disease and rely on numerical datasets, which reduces their effectiveness in early-stage prediction. In the practical scenarios, early warning from the first stage should have been drawn out for effective treatment. This raises the motivation of designing a Colorectal Cancer Diagnosis system based on Machine Learning that is not only effective in accuracy but also simple enough for practice.

The remaining of this paper is presented in the following sections. Section 2 presents collection method and its description. The methodology and the details of our proposed model are introduced in Section 3. Section 3 shows the experimental environment and the results of implementing the new model on specific data sets. The last section includes the conclusions and the directions for further works.

## 2. Materials

### Data description

Data of an Electronic Medical Record (EMR) is a crucial component in managing patient information and providing efficient healthcare in a hospital. The issue of using EMR data for machine learning models to support physicians in diagnosis is an important and meaningful matter. This study does not involve any human or animal participation.

EMRs can be understood as an electronic recording system containing patients’ medical and health information. It replaces traditional paper-based recording systems and patient records by storing medical information in an electronic database. An EMR includes information of medical history, test results, medical images, prescriptions, information about surgeries and treatments, contact information, and other health-related information about patients. Data is generated and updated by healthcare providers, including physicians, nurses, healthcare personnel, and other service providers. However, there are numerous challenges posed by the current state of EMRs, including lack of standardization, disparate data, and incomplete processing and encoding of medical record entries.

**Fig 1:**
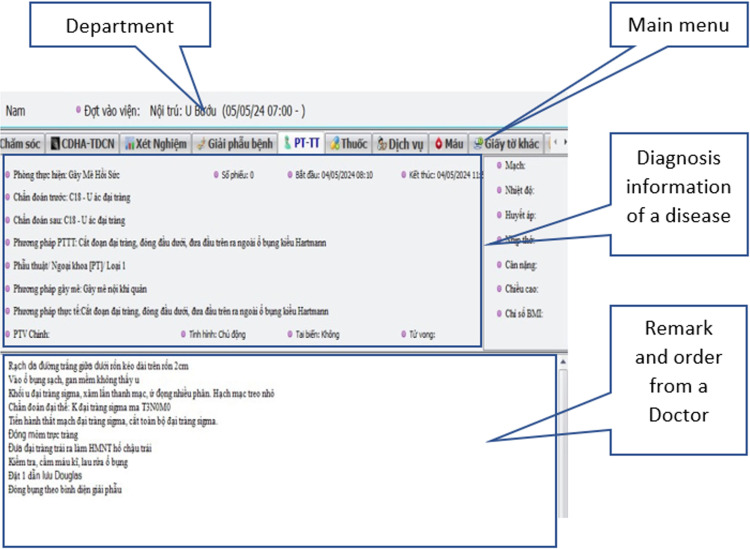
A HIS’ interface supports doctor in entering examination results.

In this study, the process of examination and diagnosis of Colorectal cancer is proposed and implemented on the data of Thai Nguyen Central Hospital under the ACCEPTANCE OF THE ETHICS COUNCIL OF THAI NGUYEN NATIONAL HOSPITAL, No. 27/HĐĐĐ-BVTWTN Jan, 10^th^ 2022. The data were accessed and collected from Jan 15, 2022 to Dec 25, 2022 based on the CERTIFICATE OF COOPERATION IN IMPLEMENTING, April, 26 2021 between Thai Nguyen National Hospital and University of Information and Communication Technology, Thai Nguyen University. Fig 1 shows the HIS system which is being used in the hospital.

Retrospective data collection method is deployed to extract information from electronic medical records of patients with gastrointestinal diseases and symptoms related to Colorectal cancer. This process is divided 4 phases:

#### The first phase: clinical examination.

A doctor will focus on exploiting the patient’s symptoms and risks.

1. Asking and checking the intestinal circulation disorders
2. Asking and check for bloody mucus
3. Examine and check for signs in the hypogastric area, the feeling of incomplete bowel movements.
4. Inquire and check for diarrhea or constipation syndrome.
5. Inquire and check for changes in stool shape.
6. The patient’s history is related to cancer risk.

#### The second phase: medical examination.

1. Examination of whole body symptoms:
2. Check hematology through blue skin color, mucous membranes, red blood cell test, hemoglobin test.
3. Weight loss - Physical symptoms
4. Rectal examination
5. Abdominal examination, checking for right colon and sigmoid colon tumors. Also, check for signs of intestinal obstruction.

#### The third phase: order paraclinical tests.

1. Endoscopy of the entire colorectal cavity.
2. Pathology and molecular biology
3. Diagnostic imaging tests such as abdominal ultrasound, CT scan, abdominal MRI of metastatic tumors in the abdomen, and chest CT to evaluate lung metastasis.
4. Tumor marker test: CEA, CA 19–9.
5. Monitoring treatment response and predict metastasis after treatment.
6. Hematology tests evaluate anemia, biochemical tests evaluate liver and kidney function.

#### The fourth phase: conclusion of diagnosis.

1. Based on the results of clinical and paraclinical examination, the doctor makes a conclusion to diagnose the disease.
2. Relying on pathology is the most important criterion to conclude cancer.

**Fig 2:**
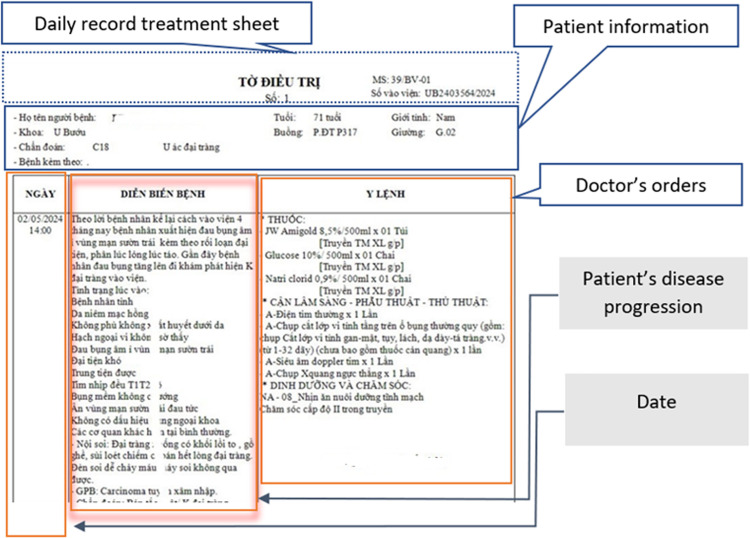
A disease progression in EMR.

An example of conclusion is given in Fig 2 below.

For data source was extracted from the electronic medical records of patients who were examined and treated in two departments: Gastroenterology and Oncology.

• Sample selection criteria: *Patients exhibiting symptoms of Colorectal cancer who consented to participate in the study.*
• Exclusion criteria: *Patients with psychiatric disorders or unstable psychological conditions, patients with untreated unstable systemic illnesses, patients who do not consent to participate in the study.*

**Fig 3:**
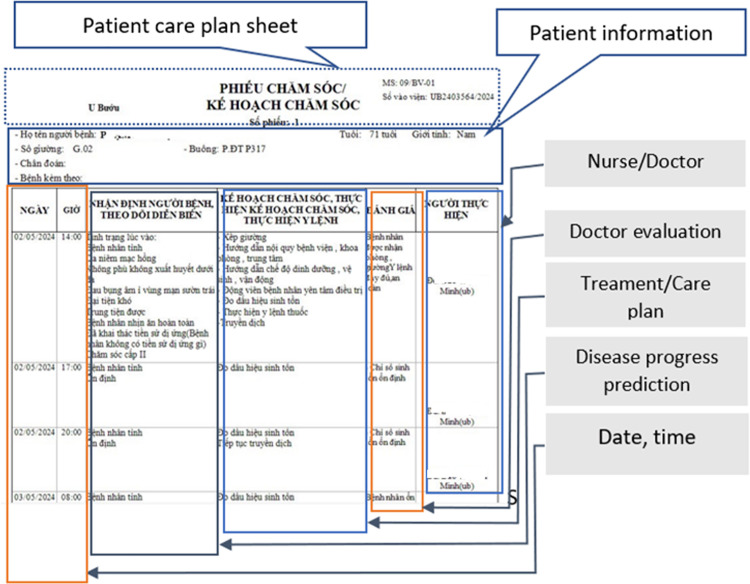
Patient Care Plan Sheet with Doctor’s Evaluation and Treatment Notes.

All data collected are encoded and ensure the confidentiality of all collected medical record entries. Fig 3 shows a part of a EMR of a patient who was visited Oncology Department.

• The collected data pertains to the keyword “Colon” for inpatients examined in the Oncology Department and the Gastroenterology Department, with patient codes D18.1 and D18.2.
• The dataset includes information collected from 1200 patients with 34 attributes, including details related to gender, age, place of residence, pre-hospitalization condition, personal medical history upon admission, etc.

From the collected attributes set, under the analysis and selection of expert physicians in the field of gastrointestinal examination and treatment, particularly in Colorectal cancer, 21 feature attributes related to diagnostic symptoms of Colorectal cancer are identified as in Table 1.

**Table 1:** List of attributes related to diagnostic symptoms of colorectal rectal cancer.

Order	Feature attributes
1	Abdominal reaction
2	Mucous membrane
3	Peritoneal irritation
4	Peripheral lymph nodes
5	Abdominal pain
6	Diarrhea
7	Slithering signs
8	Nausea
9	Lower rib pain
10	Parotid signs
11	Anorexia
12	Abdominal distention/ bloated
13	Cyanotic skin
14	Constipation
15	Stool (with blood)/ blood in stool
16	Colonoscopy
17	Abdominal computed tomography/ abdominal CT (CT)
18	Supraclavicular lymph nodes
19	Lose weight
20	Fecal incontinence
21	Hyperpigmentation/ Tan

To extract important information from the 21 feature attributes, there are 3 main steps, including:

**Step 1:** The electronic text data is proceeded using a Natural Language Processing (NLP) model called Named Entity Recognition (NER). Through this step, key keywords within the mentioned 21 attributes are defined.
**Step 2:** Data filtering is conducted by the research team to identify key keywords.
**Step 3:** The accuracy of the NER in step 1 and step 2 is evaluated. The NER (Named Entity Recognition) model is built to learn how to classify words in text into named entity labels. Common models used in NER include Conditional Random Fields (CRF) models, LSTM-CRF models, and BERT models. The accuracy of the NER model is evaluated using metrics such as Precision, Recall, and F1-score.

## 3. Methods

**Fig 4:**
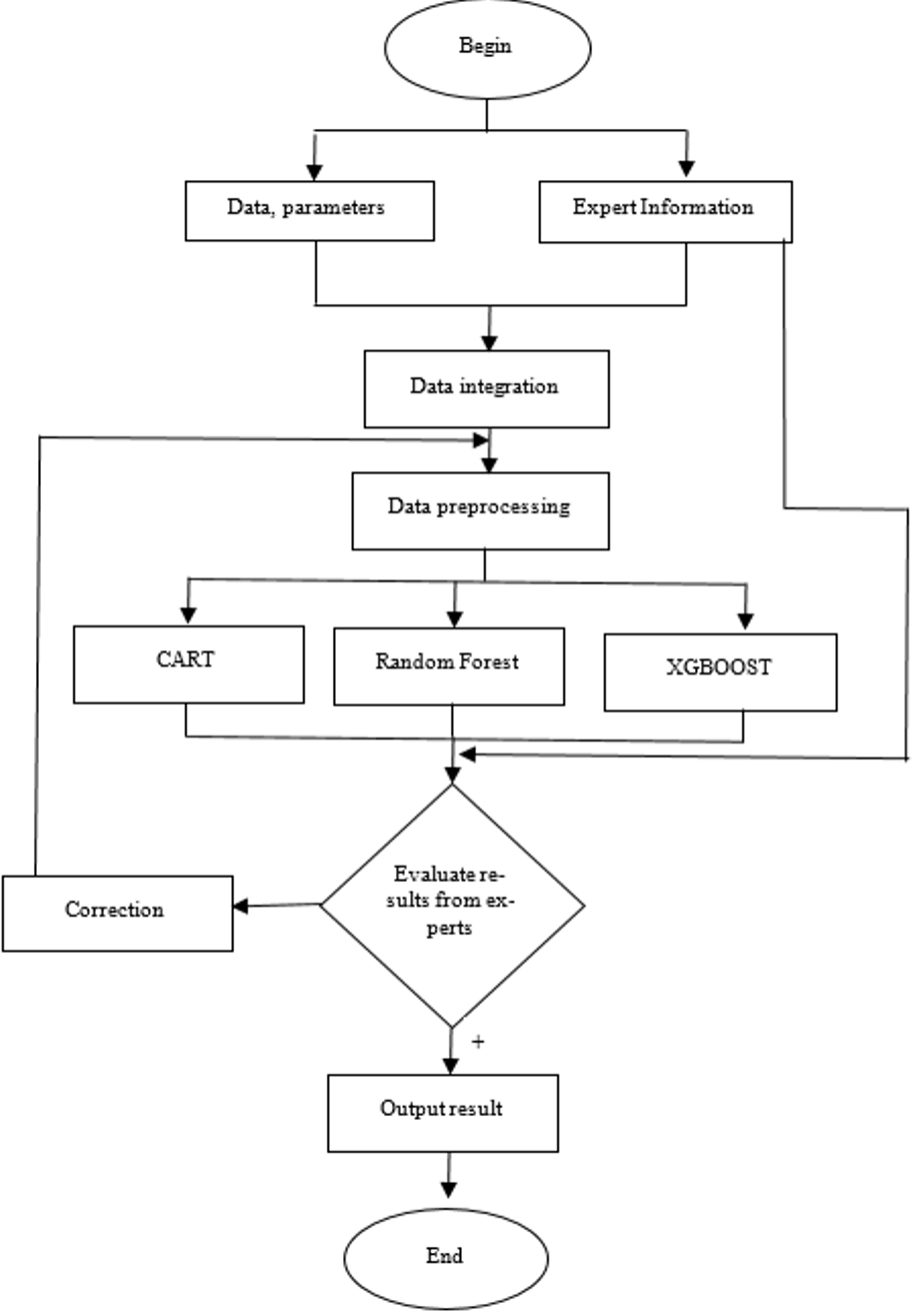
Machine learning model in supporting Colorectal cancer diagnosis.

Based on the data collected and through the preprocessing steps in Section 2 and also results of the analysis on previous studies of the machine learning models, including CART, Random Forest, XGBOOTS in Section 1. A novel method combining various machine learning methods to support development of diagnosis of Colorectal cancer system is proposed. Fig 4 presents the architecture of the proposed system.

Its implementation can describe through 6 steps as below:

**Step 1:** Collecting and integrating features clinical data.

**Fig 5:**
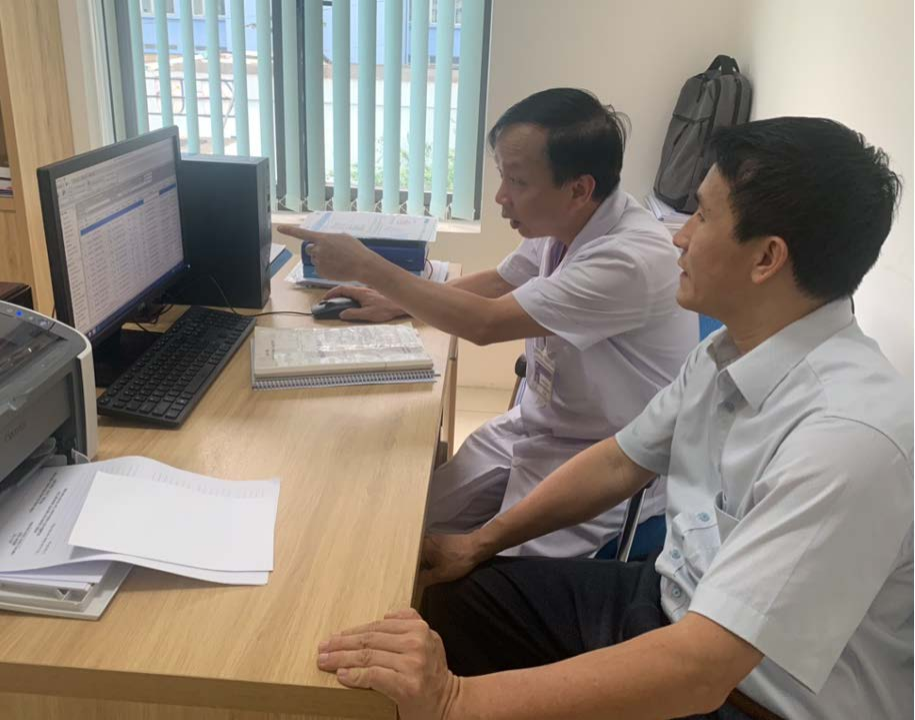
An expert was explaining his evaluations.

In this step, some surveys and discussions with medical specialists are performed in order to determine detailed data requirements for the Colorectal cancer problem. Then, based on the received information, the features of data are collected from the Department of Oncology, Department of Internal Medicine and General Surgery, ensuring that the completeness and quality of data meet the requirements. This study was reviewed and approved by the institutional review board (ethics committee) of Thai Nguyen National Hospital, Vietnam. We have included the confirmation letter. All patients have been interviewed and images have been collected with their consents. The individual pictured in Fig 5 has provided written informed consent to publish their image alongside the manuscript.

**Step 2:** *Revising the features by asking experts.*

Using the collected attribute data, the questions are asked to some experts of oncology to take the most typical attributes of Colorectal cancer so that machine learning model can be used.

**Step 3:** *Cleaning data and standardization of terminologies*

Based on received data on step 1 and expert’s recommendations, data is cleaned and the terminology is upgraded in term of medicine so that the data is became the most possible in real.

**Step 4:** *Adapting data to the model*

The cleaned data is transformed into secondary data as input for the machine learning model.

**Step 5:** *Implementation*

Applying CART, Random Forest, XGBOOT algorithms to mine data and build prediction models.

**Step 6:** *Evaluation*

Using the data, the accuracy of model is evaluated and compared to the specialist’s evaluation. From the comparison results, the ability of applying the diagnosis in real situation is considered. Fig 5 shows you the way an expert explained what he needs from system. The individual pictured in Fig 5 has provided written informed consent to publish their image alongside the manuscript.

To ensure the accuracy and reliability of the data and model results, each step may need to be iteratively refined. In such cases, close collaboration with oncologists is essential to enhance data quality and adjust the model, ensuring that the methodology aligns with expert knowledge and clinical practices in disease treatment.

### Ethics statement

This research does not involve any human or animal participation. This study was reviewed and approved by the institutional review board (ethics committee) of Thai Nguyen National Hospital, Vietnam. We have included the confirmation letter. All patients have been interviewed and images have been collected with their consents. The individual pictured in Fig 5 has provided written informed consent to publish their image alongside the manuscript.

## 4. Results and discussion

### 4.1. Experimental setup

For data processing, analysis, and visualization, the following libraries were applied: PANDAS, SKLEARN, XLSXWRITER, MATH, MATPLOTLIB, and PYVI using an Asus laptop with an Intel Core i5-10300H processor, 8GB RAM, and the Ubuntu 20.04 operating system.

The dataset used in this research was collected from patients diagnosed and treated for colorectal cancer. There are 21 symptoms related to rectal cancer. Because of some noises in data, it’s necessary to preprocess obtained dataset. Library PYVI is used to omit punctuations.

The standardized data is mapped in form of values in {0, 1}. In which, if any symptom is available in patients, the value of this attribute is assigned by 1. Otherwise, the value of attribute is 0. The same task is performed on the diagnosis column. The samples of patients with conclusion as Colorectal cancer are marked as 1. The samples of other patients are marked as 0.

The progress of preprocessing data includes following steps

- Standardize diagnosis dataset into “cancer” or “no cancer”. Some other cases, expert knowledge is necessary.
- Normalize the words in dataset by eliminating unnecessary space, correcting the typos.
- Select the most important symptoms, remove the symptoms that unrelated to rectal cancer.
- Consult oncologists for patients who have not been diagnosed yet.
- Filter the typical symptoms related to Colorectal cancer from oncologists.

### 4.2. Experimental results

The experimentation was deployed on an independent data set of 400 EMRs. Data set for testing is also subjected to the same operations as the model training data set. After combining this symptom information with the decision tree, a set of predictions about the patient’s health situation is released. Furthermore, results of the diagnostic were double checked with the doctor’s diagnostic results and then the model’s performance parameters will be determined.

The implementations are performed in 3 different scenarios:

- Scenario 1 (Kb1): 80% of the data is used for training and 20% is used for testing model.
- Scenario 2 (Kb2): 70% of the data is used for training and 30% is used for testing model.
- Scenario 3 (Kb3): 6% of the data is used for training and 40% is used for testing model.

The experimental results are shown in Table 2.

**Table 2:** Implementation results.

Models	Accuracy			Precision			Recall			F1 Score		
	Kb1	Kb2	Kb3	Kb1	Kb2	Kb3	Kb1	Kb2	Kb3	Kb1	Kb2	Kb3
CART	0.9516	0.9469	0.9501	0.9731	0.9701	0.9717	0.9760	0.9739	0.9760	0.9745	0.9720	0.9739
Random Forest	0.9573	0.9469	0.9487	0.9732	0.9663	0.9688	0.9820	0.9780	0.9775	0.9776	0.9721	0.9732
XGBOOST	0.9573	0.9507	0.9516	0.9676	0.9646	0.9717	0.9880	0.9840	0.9775	0.9777	0.9742	0.9746

As shown in Table 2, the performance of selected models is equivalent. The measurements used in our assessment include Accuracy, Precision, Recall and F1 Score, same as in [21]. The detail comparison in each measurement can be stated as:

- Accuracy: XGBOOST and Random Forest have equivalent results in scenario 1 (95.73%), but XGBOOST is outperform in scenarios 2 and 3. Thus, among the 3 methods, XGBOOST gives better results. CART has stable results in scenarios reaching about 95%.
- Precision: Random Forest has the best accuracy in scenario 1, while CART has the best performance in scenarios 2 and 3 (more than 97%). CART and XGBOOST have the best accuracy in scenario 3 (97.17%).
- Recall: XGBOOST has the best performance in all three scenarios in term of Recall. The results of Random Forest are a bit higher than that of CART in three scenarios.
- F1 Score: XGBOOST maintains the top position with the highest F1 score in all three scenarios (above 97.4%). Random Forest is light better than CART in scenarios 1 and 2.

XGBOOST is an excellent choice for this problem with good performance on many measures. Random Forest and CART also have good performance, but not as superior as XGBOOST.

## 5. Conclusions

In this paper, a real dataset from a hospital is collected and preprocessed. Apart from that, a novel method is proposed. This model combines unified academic algorithms to support Colorectal cancer prediction. The article has main contributions as follows: (i) Appling learning models in the problem of supporting Colorectal cancer prediction; (ii) Proposing a method to collect data of Colorectal cancer from EMRs and then train the data to use it in learning models; (iii) Experimental results based on 4 measures Accuracy, Precision, Recall, F1 Score also selected the XGBOOST model for better results than some CART and Random Forest methods.

According to experts, with this approach when the system is complete, it will provide good support for doctors in phase 1 and phase 2 of the Colorectal cancer examination process.

**Fig 6:**
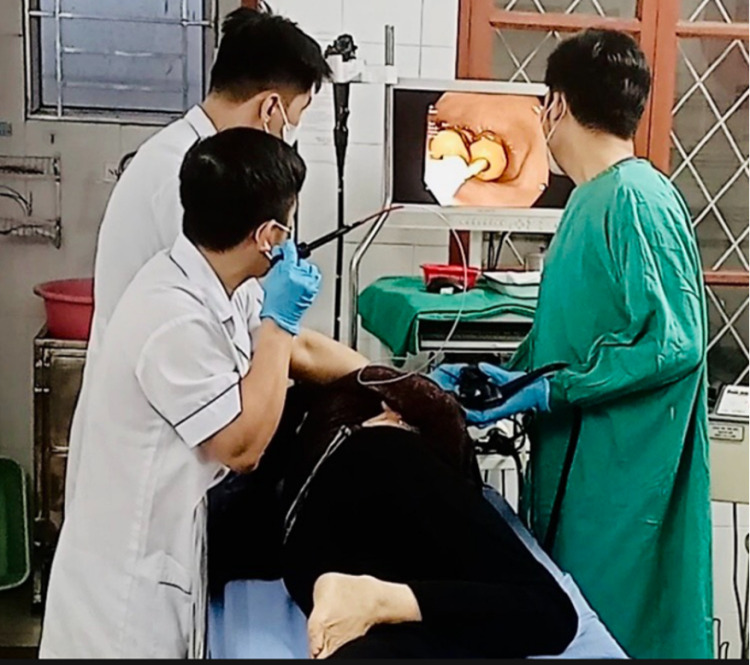
Doctors was taking a colonoscopy.

Also, the system will support a doctor how to minimize redundant prescriptions of tests and scans in phase 3 thereby it will support a doctor quickly reach a final conclusion on whether the patient has Colorectal cancer or not. Because of this, a patient can reduce waiting time and costs when doing test procedures. Fig 6 shows you doctors took a colonoscopy.

Finally, based on the obtained results, we believe that this method will create a foundation for further research in applying machine learning models to support solving some practical problems in medicine.
