# Expression of Concern: A practical approach for colorectal cancer diagnosis based on machine learning

**DOI:** 10.1371/journal.pone.0343787

**Published:** 2026-02-26

**Authors:** The PLOS One Editors

After this article [1] was published, the following concerns were noted:

- The articles cited as References 2 and 12 were retracted prior to publication of [1].
- Several references do not appear to support the statements for which they were cited, including References 1, 2, and 4-12.
- Several statements in the introduction are not supported by references.
- The article does not comply with the PLOS Data Availability policy.

The corresponding author acknowledged the citation issues and provided alternatives for the two retracted references; however, the Editors determined that the replacements did not adequately support the cited statements.

The corresponding author stated that the data cannot be provided due to patient confidentiality and institutional regulations. The Editors consider this to be sufficient to meet the requirements of the data policy related to sensitive patient data.

The Data Availability statement is updated to: Requests for data access may be directed to the Head of the Information Technology Department of Thai Nguyen National Hospital via cntt@bvdktuthainguyen.gov.vn.

In light of the unresolved reference issues, the *PLOS One* Editors issue this Expression of Concern.

## References

[1] Hai Minh N, Quy TQ, Tam ND, Tuan TM, Son LH. A practical approach for colorectal cancer diagnosis based on machine learning. PLoS One. 2025;20(4):e0321009. doi: 10.1371/journal.pone.0321009 40300028 PMC12040227

